# Advantages of adjuvant chemotherapy for patients with triple-negative breast cancer at Stage II: usefulness of prognostic markers E-cadherin and Ki67

**DOI:** 10.1186/bcr3068

**Published:** 2011-11-30

**Authors:** Shinichiro Kashiwagi, Masakazu Yashiro, Tsutomu Takashima, Naoki Aomatsu, Katsumi Ikeda, Yoshinari Ogawa, Tetsuro Ishikawa, Kosei Hirakawa

**Affiliations:** 1 Department of Surgical Oncology, Osaka City University Graduate School of Medicine, 1-4-3 Asahi-machi, Abeno-ku, Osaka, Japan; 2 Oncology Institute of Geriatrics and Medical Science, Osaka City University Graduate School of Medicine, 1-4-3 Asahi-machi, Abeno-ku, Osaka, Japan; 3 Department of Breast-surgical Oncology, Osaka City General Hospital, 2-13-22 Miyakojima-hondori, Osaka, Japan

**Keywords:** chemosensitivity, E-cadherin, Ki67, predictive marker, triple-negative breast cancer

## Abstract

**Introduction:**

Triple-negative breast cancer (TNBC), which is characterized by negativity for estrogen receptor, progesterone receptor and human epidermal growth factor receptor 2 (HER2), is a high risk breast cancer that lacks specific targets for treatment selection. Chemotherapy is, therefore, the primary systemic modality used in the treatment of this disease, but reliable parameters to predict the chemosensitivity of TNBC have not been clinically available.

**Methods:**

A total of 190 TNBC patients who had undergone a curative resection of a primary breast cancer were enrolled. The adjuvant chemotherapy was performed for 138 (73%) of 190 TNBC cases; 60 cases had an anthracyclin-based regimen and 78 a 5-fluorouracil-based regimen. The prognostic value of E-cadherin, Ki67 and p53 expression in the outcome of TNBC patients with adjuvant chemotherapy was evaluated by immunohistochemistry.

**Results:**

The adjuvant therapy group, especially those with Stage II TNBC, had a more favorable prognosis than the surgery only group (*P *= 0.0043), while there was no significant difference in prognosis between the anthracyclin-based regimen and 5-fluorouracil-based regimen. Patients with E-cadherin-negative and Ki67-positive expression showed significantly worse overall survival time than those with either E-cadherin-positive or Ki67-negative expression (*P *< 0.001). Multivariate analysis showed that the combination of E-cadherin-negative and Ki67-positive expression was strongly predictive of poor overall survival (*P *= 0.004) in TNBC patients receiving adjuvant chemotherapy. In contrast, p53 status was not a specific prognostic factor.

**Conclusions:**

Adjuvant therapy is beneficial for Stage II TNBC patients. The combination of E-cadherin and Ki67 status might be a useful prognostic marker indicating the need for adjuvant chemotherapy in Stage II TNBC patients.

## Introduction

Breast cancer is a heterogeneous disease and is currently divided into subtypes in accordance with the status of estrogen receptor (ER), progesterone receptor (PR) and human epidermal growth factor receptor 2 (HER2) [1-3]. These subtypes display significant diversity in regard to the clinical behavior, outcome and response to therapy [4-6]. One of these subtypes, triple-negative breast cancer (TNBC), which is characterized by a lack of ER, PR and HER2 expression, accounts for 10% to 20% of all breast cancers, and has a high probability of early tumor relapse after diagnosis, increased propensity to develop brain metastases, and rapid risk of death after tumor relapse [1,7-9]; adjuvant therapy is thus necessary for patients with TNBC [10]. However, since TNBC lacks specific targets for treatment selection, chemotherapy is the primary systemic modality used in the treatment of this disease [11].

A recent study has demonstrated that TNBC is more chemosensitive than other subtypes of breast cancer [12]. Kennedy *et al. *reported that patients with TNBC who underwent adjuvant chemotherapy were 52% less likely to die compared with those who received neoadjuvant chemotherapy or no/unknown chemotherapy [13], suggesting that the benefit of primary tumor removal followed by early initiation of adjuvant therapy may be most relevant for the TNBC subgroup. Anthracyclines (epirubicin and doxorubicin), alkylating agents (cyclophosphamide), and 5-fluorouracil (5FU) are the standard of care in the treatment of breast cancer in the adjuvant setting.

The selection of patients with chemosensitive tumors before initiating chemotherapy would be important for avoiding potential therapy-related complications. Predictive factors of response would help to assess the expected individual benefit of this treatment. Different breast cancer subgroups may have different predictive markers of response to chemotherapy. Thus, it is of the highest importance to elucidate prognostic factors and key biomarkers of triple-negative cancers. Although various *in vivo *and *in vitro *approaches have been used in an attempt to predict the chemosensitivity of TNBC [14-16], reliable parameters have not been clinically available. The purpose of this study was to evaluate candidate predictive markers for chemosensitivity in TNBC.

E-cadherin, one of the cell adhesion molecules, is reported to be related to the invasion of cancer cells, and a low-level expression of E-cadherin is considered to be an indication of poor prognosis [17-22]. Although E-cadherin is one of the markers for chemosensitivity in several types of carcinomas [23-25], the significance of E-cadherin for chemosensitivity of TNBC remains unclear [25]. Ki67 has been reported to be a candidate predictive marker for chemosensitivity in all types of breast cancer [16,26], but the predictive value of Ki67 for chemoresponse of TNBC has not been clarified. p53 status is one of the most investigated predictive biomarkers for the efficacy of anthracycline-containing chemotherapy. Despite the many studies, however, the results have been inconsistent, with some studies reporting an association between p53 expression and tumor response to neoadjuvant anthracyclines [27-29], whereas other reports have associated p53 overexpression with both resistance [30,31] and sensitivity [32,33] to preoperative anthracycline-containing chemotherapy. TNBC is more likely to carry TP53 gene mutations. In the present study, we evaluated the prognostic value of E-cadherin, Ki67 and p53 expression for the outcome of adjuvant chemotherapy in 190 cases of TNBC, which were culled from 1,036 cases of all types of breast carcinomas.

## Methods

### Patients

This study investigated a consecutive series of 1,063 cases of sporadic invasive breast carcinoma. Because E-cadherin is functionally silenced in invasive lobular carcinoma [34], 27 cases of invasive lobular carcinomas were excluded. Then a total of 1,036 breast cancer cases were enrolled in this study. All patients had received a curative operation of a mastectomy or a conservative surgery with axillary lymph node dissection in Osaka City University Hospital or Osaka City General Hospital from 2000 to 2006. The median follow-up time was 3.6 years (range, 0.2 to 6.0 years). Tumors were confirmed histopathologically and staged according to the TNM classification [35]. All patients who underwent breast-conserving surgery were administered post-operative radiotherapy. Adjuvant chemotherapy was performed either by an anthracycline-based regimen (doxorubicin or epirubicin) or by a 5FU-based regimen in TNBC depending on the stage or risk of recurrence in accordance with the National Comprehensive Cancer Network (NCCN) guidelines [36]. This study was conducted with the consent of the ethical committee of Osaka City University, and informed consent was obtained from all subjects. Overall survival time was set in days as the period from the initial surgery.

### Immunohistochemistry

**Figure 1:**
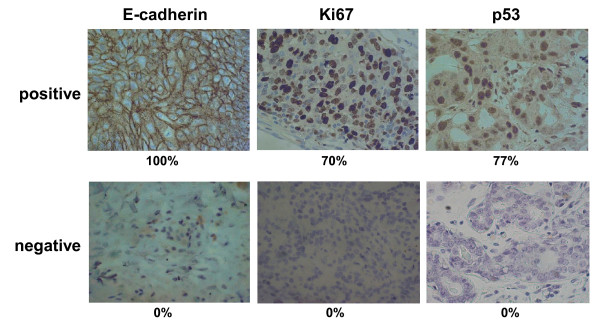
**Immunohistochemical determination of E-cadherin, Ki67, and p53**. E-cadherin was observed at cell-cell boundaries of breast cancer cells. Ki67 and p53 were found in the nuclei of cancer cells. E-cadherin, Ki67 and p53 expressions were evaluated by percentage of stained tumor cells. The upper paragraph showed positive-staining of E-cadherin (100%), Ki67 (70%) and p53 (77%), and the lower paragraph showed as negative-staining (0%) as score 0.

Immunohistochemical study was performed as previously reported [37]. Briefly, the formalin embedded tissue sections were deparaffinized, and were heated in Target Retrieval Solution (Dako, Carpinteria, CA, USA). Sections were then incubated in 10% normal goat or rabbit serum to reduce non-specific antibody binding. Tissue sections were then incubated with each primary monoclonal antibody against ER (clone 1D5, dilution 1:80; Dako, Cambridge, UK), PR (clone PgR636, dilution 1:100; Dako), HER2 (Hercep Test, Dako), p53 (clone DO-7, dilution 1:50; Dako), Ki67 (clone MIB-1, dilution 1:00; Dako) and E-cadherin (clone NCH-38, dilution 1:200; Dako). The slides were treated with streptavidin-peroxidase reagent, and were incubated in PBS diaminobenzidine and 1% hydrogen peroxide v/v, followed by counterstaining with Mayer's hematoxylin. Positive and negative controls for each marker were used according to the supplier's data sheet (Dako). Immunohistochemical scoring was performed in a blind fashion. The cut-off for ER positivity and PR positivity was > 0% positive tumor cells with nuclear staining. HER2 was graded according to the accepted grading scheme as 0, 1+, 2+, 3+. the following criteria were used for scoring: 0, no reactivity or membranous reactivity in less than 10% of cells; 1+, faint/barely perceptible membranous reactivity in 10% of cells or higher or reactivity in only part of the cell membrane; 2+, weak to moderate complete or basolateral membranous reactivity in 10% of tumor cells or higher; 3+, strong complete or basolateral membranous reactivity in 10% of tumor cells or higher. HER-2 was considered to be positive if immunostaining was 3+ or if a 2+ result showed gene amplication by fluorescent *in situ *hybridization (FISH). In FISH analyses, each copy of the *HER2 *gene and its centromere 17 (*CEP17*) reference were counted. The interpretation followed the criteria of the ASCO/CAP guidelines for HER2 IHC interpretation for breast cancer [38]: positive if the *HER2*/CEP17 ratio was higher than 2.2. The cut-off for p53 was ≥ 1% positive tumor cells with nuclear staining. Ki67-labelling index > 30% was determined to be positive. E-cadherin antibody intensely stained the membrane and weakly stained the cytoplasm of cancer cells. E-cadherin expression was semi-quantitatively analyzed according to the percentage of cells showing membrane positivity: 0, 0 to 10%; 1+, 10 to 30%; 2+, 30 to 70%; 3+, > 70%. E-cadherin expression was considered positive when scores were ≥ 2, and negative when scores were ≤ 1. Cytoplasmic staining only was not included in the assessment. E-cadherin antibody intensely stained the membrane and weakly stained the cytoplasm of cancer cells. A case with cytoplasmic staining only was determined as E-cadherin negative, as shown with "score 0" (Figure 1).

### Statistical analysis

Statistical analysis was performed using SPSS 13.0 statistical software (SPSS Inc, Chicago, IL, USA). We examined the association between TNBC and other clinicopathologic variables, and the significance of different prognostic markers using chi-squared test, and chi-squared test for trend as appropriate. The association with survival was analyzed initially by Kaplan-Meier plot and log-rank test and also with Cox regression analysis to adjust for other prognostic indicators. A *P*-value of < 0.05 was considered significant. Cutoff values for different biomarkers included in this study were chosen before statistical analysis.

## Results

### The prognostic value of adjuvant chemotherapy in triple-negative breast cancer

**Figure 2:**
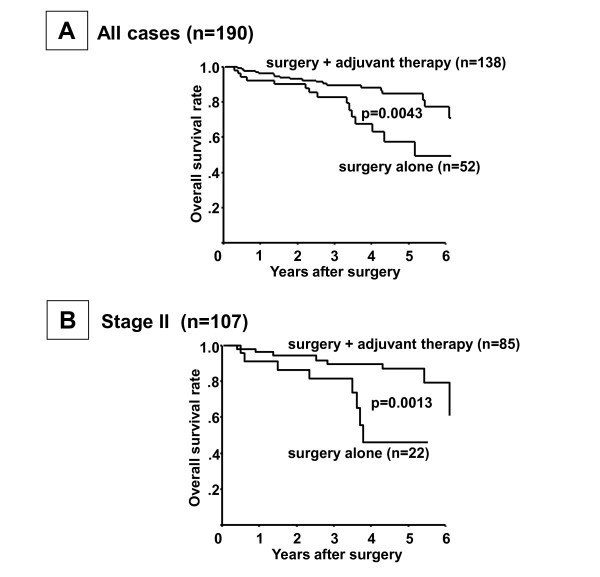
**Overall survival of patients with triple-negative breast cancer according to receipt of adjuvant therapy**. **(A) **Patients who underwent surgery plus adjuvant therapy showed a better prognosis than those who underwent surgery alone (*P *= 0.0043). **(B) **Prognosis of patients according to the clinical stage. Overall survival of patients by adjuvant chemotherapy was significantly better at Stage II (*P *= 0.0013).

Cases that were negative for ER, PR and HER2 expression were considered to be cases of TNBC. Among the total 1,036 breast cancer cases, there were 190 (18.3%) cases of TNBC. Adjuvant chemotherapy was performed for 138 (72.6%) of the 190 TNBC cases; 60 cases had an anthracyclin-based regimen and 78 a 5FU-based regimen (Table 1). The remaining 52 cases received surgery alone. Among the 190 TNBC cases, those receiving surgery plus adjuvant therapy (*n *= 138) had a more favorable prognosis (*P *= 0.0043) than those undergoing surgery alone (*n *= 52) (Figure 2a). When restricting the analysis to patients with Stage II cancers, the overall survival of the surgery plus adjuvant chemotherapy group was significantly better than that of the surgery alone group (*P *= 0.0013) (Figure 2b). In contrast, in patients with Stage I and III cancers, no significant difference of overall survival was found between the surgery plus adjuvant chemotherapy group and the surgery alone group (Additional file 1). There was no significant difference in the prognosis of TNBC patients receiving adjuvant therapy between the anthracyclin-based regimen and 5FU-based regimen (Table 2).

**Table 1:** Regimen of chemotherapy in triple-negative breast cancers

Adjuvant chemotherapy regimen	Number of patients
Anthracyclin-base	60
fluorouracil+epirubicin+cyclophosphamide (FEC)	34
epirubicin+cyclophosphamide (EC)	12
adriamycin+cyclophosphamide (AC)	11
cyclophosphamide+adriamycin+fluorouracil (CAF)	3
non-Anthracyclin-base	78
tegafur-uracil (UFT)	61
cyclophosphamide+methotorexate+fluorouracil (CMF)	10
doxifluridine (5'DFUR)	4
fluorouracil (5FU)	3

**Table 2:** Univariate and multivariate analysis with respect to overall survival in 190 triple-negative breast cancers

	Univarite analysis			Multivariate analysis		
		
Parameters	Odds ratio	95% CI	*P-*value	Odds ratio	95% CI	*P-*value
Regimen Anthracyclin-base vs 5FU-base	0.38	0.13 to 1.08	0.068	0.46	0.15 to 1.38	0.164
E-cadherin						
Negative vs Positive	0.25	0.09 to 0.72	0.010	0.26	0.09 to 0.76	0.013
Ki67						
Negative vs Positive	2.38	1.14 to 4.99	0.022	2.11	0.96 to 4.63	0.062
E-cadherin (-) and Ki67 (+)						
Negative vs Positive	3.03	1.54 to 5.98	0.001	2.78	1.38 to 5.60	0.004
p53						
Negative vs Positive	1.60	0.75 to 3.42	0.229	1.13	0.51 to 2.52	0.758
Stage						
1 vs 2, 3, 4	2.54	1.04 to 6.22	0.041	0.30	0.05 to 2.00	0.214
Tumor size						
≤ 2 cm vs > 2 cm	2.46	1.11 to 5.45	0.027	3.25	0.75 to 14.15	0.116
Lymph node status						
N0 vs N1, N2, N3	3.40	1.68 to 6.91	0.001	3.46	1.38 to 8.66	0.008
Lymph-vascular invasion						
Negative vs Positive	1.84	0.94 to 3.58	0.074	1.36	0.68 to 2.74	0.390
Nuclear grade						
1, 2, vs 3	2.36	1.07 to 5.21	0.034	1.93	0.58 to 6.39	0.282

### Significance of E-cadherin, Ki67, and p53 expression in triple-negative breast cancer

Expression of E-cadherin, Ki67, and p53 was positive in 109 (57%), 65 (34%) and 118 (62%) of 190 cases of TNBC, respectively (Table 3). The Ki67 expression level was significantly high in Stage II and III TNBC tumors (63%, *P *= 0.013). No significant association between E-cadherin or p53 expression and clinicopathological parameters was identified in TNBC. The cases with E-cadherin-negative and Ki67-positive expression had a significantly higher incidence of lymph node metastasis (45%, *P *= 0.027).

**Table 3:** Correlation between clinicopathological features and Ki67, E-cadherin, and p53 expression in 190 triple-negative breast cancers

	Ki67		*P-v*alue	E-cadherin		*P-*value	Ki67 (+) and E-cadherin (-)		*P-*value	p53		*P-v*alue
								
Parameters	Positive	Negative		Positive	Negative		Positive	Negative		Positive	Negative	
	(*n *= 109)	(*n *= 81)		(*n *= 65)	(*n *= 125)		(*n *= 68)	(*n *= 122)		(*n *= 118)	(*n *= 72)	
Age at operation												
≤ 55	49 (58%)	35 (42%)		29 (35%)	55 (65%)		31 (37%)	53 (63%)		57 (68%)	27 (32%)	
> 55	60 (57%)	46 (43%)	0.811	36 (34%)	70 (66%)	0.935	37 (35%)	69 (65%)	0.775	61 (57%)	45 (43%)	0.146
Pathological stage												
I	26 (44%)	33 (56%)		20 (34%)	39 (66%)		16 (27%)	43 (73%)		36 (61%)	23 (39%)	
II and III	83 (63%)	48 (37%)	0.013	45 (34%)	86 (66%)	0.951	52 (40%)	79 (60%)	0.094	82 (63%)	49 (37%)	0.836
pTumor size												
≤ 2 cm	37 (49%)	38 (51%)		24 (32%)	51 (68%)		26 (35%)	49 (65%)		45 (60%)	30 (40%)	
> 2 cm	72 (63%)	43 (37%)	0.071	41 (36%)	74 (64%)	0.604	42 (37%)	73 (63%)	0.794	73 (63%)	42 (37%)	0.629
pLymph node status												
Negative	60 (52%)	55 (48%)		43 (37%)	72 (63%)		34 (30%)	81 (70%)		70 (61%)	45 (39%)	
Positive	49 (65%)	26 (35%)	0.073	22 (31%)	53 (69%)	0.252	34 (45%)	41 (55%)	0.027	48 (64%)	27 (36%)	0.664
Lymph-vascular invasion												
Negative	72 (54%)	60 (46%)		49 (37%)	83 (63%)		43 (33%)	89 (67%)		79 (60%)	53 (40%)	
Positive	37 (64%)	21 (36%)	0.235	16 (28%)	42 (72%)	0.202	25 (43%)	33 (57%)	0.163	39 (67%)	19 (33%)	0.333
Histologic type												
Invasive ductal carcinoma	96 (58%)	68 (42%)		60 (37%)	104 (63%)		58 (35%)	106 (65%)		102 (62%)	62 (38%)	
Special type	13 (50%)	13 (50%)	0.414	5 (19%)	21 (81%)	0.083	10 (38%)	16 (62%)	0.76	16 (61%)	10 (39%)	0.949
Nuclear grade	46 (42%)	37 (46%)		33 (51%)	50 (40%)		27 (40%)	56 (46%)		45(38%)	38 (53%)	
1,& 2,	63 (58%)	44 (54%)		32 (49%)	75 (60%)		41 (60%)	66 (54%)		73 (62%)	34 (47%)	
3			0.633			0.156			0.409			0.048

**Figure 3:**
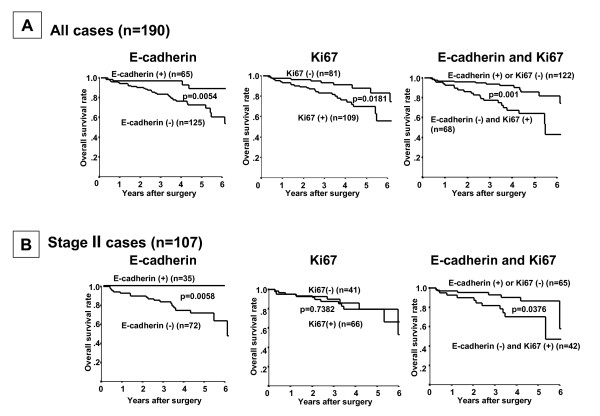
**Survival of patients with triple-negative breast cancer based on E-cadherin and Ki67 expression**. **(A) **TNBC with reduced E-cadherin expression showed a significantly worse overall survival time (*P *= 0.0054), and cases with Ki67 expression showed significantly worse overall survival time (*P *= 0.0181). Patients with E-cadherin-negative and Ki67-positive expression showed a significantly worse overall survival time (*P *= 0.001). **(B) **The prognosis of the E-cadherin-negative cancer patients was significantly poorer than that of the E-cadherin-positive cancer patients in regard to overall survival (*P *= 0.0058) in Stage II. The prognosis of the combination of E-cadherin-negative and Ki67-positive expression cancer patients was significantly poorer than that of the combination of E-cadherin-positive and Ki67-negative expression cancer patients in regard to overall survival and in Stage II (*P *= 0.0376).

TNBC with reduced E-cadherin expression showed a significantly worse overall survival time (*P *= 0.0054, log-rank), and cases with Ki67 expression showed significantly worse overall survival time (*P *= 0.0181, log-rank) (Figure 3a). Patients with E-cadherin-negative and Ki67-positive expression showed a significantly worse overall survival time (*P *= 0.001, log-rank). The prognosis of the E-cadherin-negative cancer patients was significantly poorer than that of the E-cadherin-positive cancer patients in regard to overall survival at Stage II (*P *= 0.0058) (Figure 3b). The prognosis of the combination of E-cadherin-negative and Ki67-positive expression cancer patients was significantly poorer than that of the combination of E-cadherin-positive and Ki67-negative expression cancer patients in regard to overall survival at Stage II (*P *= 0.0376) (Figure 3b) and Stage I (*P *= 0.0437) (Additional file 2). The combination of E-cadherin-negative and Ki67-positive expression was revealed to be a more significant prognostic indicator than either E-cadherin or Ki67 expression alone by multivariate analyses (*P *= 0.004, OR = 2.784), while a univariate analysis revealed that the overall survival was significantly correlated with the E-cadherin expression, Ki67 expression, stage, tumor size and lymph node metastasis (Table 2).

**Figure 4:**
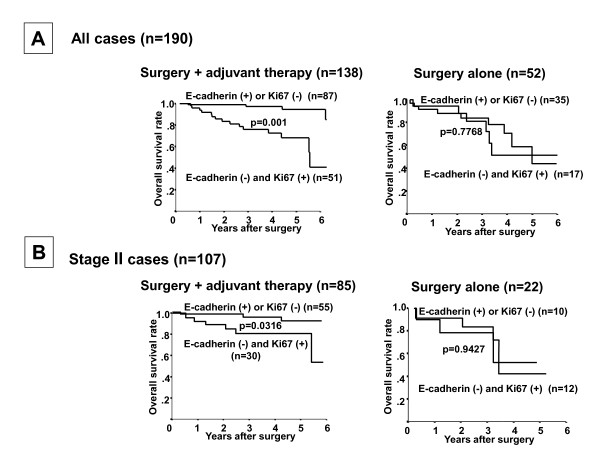
**Significance of E-cadherin and Ki67 expression in patients with or without adjuvant therapy**. **(A) **In 138 patients who underwent adjuvant therapy, the Kaplan-Meier survival curve showed that a statistically significant difference in the survival was observed in relation to the Ki67 expression and/or E-cadherin expression (*P *= 0.001). In contrast, no significant difference of prognosis in relation to the Ki67 expression and/or E-cadherin expression was found in 52 cases without adjuvant chemotherapy (*P *= 0.7768). **(B) **The prognosis of E-cadherin-negative and Ki67-positive patients was significantly poorer than that of the E-cadherin-positive or Ki67-negative patients at Stage II (*P *= 0.0316). In contrast, no significant difference of prognosis in relation to the Ki67 expression and/or E-cadherin expression was found in 22 cases without adjuvant chemotherapy at Stage II (*P *= 0.9427).

In the 138 TNBC cases undergoing surgery plus adjuvant chemotherapy, the prognosis of patients with E-cadherin-negative plus Ki67-positive expression was significantly worse that the prognosis of patients with either of these risk factors alone (*P *= 0.001), while no significant difference in prognosis was found in the 52 TNBC cases undergoing surgery alone (Figure 4a). No significant difference in prognosis was found between the E-cadherin-negative and Ki67-positive cases undergoing surgery plus adjuvant chemotherapy (*n *= 51) and surgery alone cases. A multivariate logistic regression analysis showed that the combination of E-cadherin-negative and Ki67-positive expression was significantly correlated with the overall survival of patients undergoing surgery plus adjuvant chemotherapy (*P *= 0.001), but not in those undergoing surgery alone, suggesting that E-cadherin-negative and Ki67-positive expression is an independent prognostic factor for TNBC with adjuvant chemotherapy (Table 4). In the adjuvant chemotherapy group, the overall survival of TNBC patients having both E-cadherin-negative and Ki67-positive expression was significantly worse than that of patients with either risk factor alone at each of Stages II (Figure 4), I and III (Additional file 3). In contrast, no significant difference in prognosis in relation to the Ki67 expression and/or E-cadherin expression was found in 52 cases without adjuvant chemotherapy at each of Stages II (Figure 4), I and III (Additional file 3).

**Table 4:** Univariate and multivariate analysis with respect to overall survival of 138 TNBC patients with surgery plus adjuvant chemotherapy or 52 TNBC patients with surgery alone

	Patients with chemotherapy (*n *= 138)							Patients without chemotherapy (*n *= 52)				
	
	Univarite analysis				Multivariate analysis			Univarite analysis			Multivariate analysis	
	
Parameters	Odds ratio	95% CI	*P-v*alue	Odds ratio	95% CI	*P-*value	Odds ratio	95% CI	p value	Odds ratio	95% CI	*P-*value
Ki67 (+) and E-cadherin (-) Negative vs Positive	6.69	2.41 to 18.62	<0.001	5.84	2.03 to 16.76	0.001	1.16	0.41 to 3.28	0.777	0.76	0.20 to 2.94	0.688
E-cadherin Negative vs Positive	0.02	0.00 to 1.16	0.060	0.00	0.00 to 1.98	0.943	1.00	0.32 to 3.16	0.998	1.53	0.44 to 5.36	0.503
Ki67 Negative vs Positive	3.09	1.11 to 8.66	0.032	2.60	0.87 to 7.76	0.086	1.81	0.62 to 5.31	0.282	1.89	0.42 to 8.50	0.407
p53 Negative vs Positive	1.61	0.58 to 4.45	0.361	0.98	0.34 to 2.85	0.975	1.61	0.58 to 4.45	0.361	1.86	0.54 to 6.45	0.327
Stage 1 vs 2, 3, 4	2.56	0.73 to 8.99	0.144	0.15	0.01 to 2.45	0.185	4.22	1.17 to 15.28	0.028	1.28	0.06 to 23.36	0.872
Tumor size ≤ 2 cm vs >2 cm	2.63	0.86 to 8.01	0.089	4.23	0.54 to 33.34	0.171	23.26	1.02 to 10.38	0.046	1.65	0.16 to 16.58	0.670
Lymph node status N0 vs N1, N2, N3	4.69	1.67 to 13.16	0.003	5.71	1.25 to 26.20	0.025	5.63	1.68 to 18.93	0.005	5.10	1.08 to 23.94	0.039
Lymphvascular invasion Negative vs Positive	2.09	0.86 to 5.07	0.103	1.34	0.52 to 3.44	0.548	1.32	0.48 to 3.63	0.597	0.94	0.29 to 3.09	0.922
Nuclear grade 1, 2, vs 3	2.87	0.94 to 8.72	0.063	1.64	0.51 to 5.28	0.405	1.22	0.39 to 3.86	0.732	0.99	0.25 to 3.96	0.988

## Discussion

Among the total 1,036 breast cancer cases, 190 (18.3%) were cases of TNBC. NCCN guidelines and the St. Gallen consensus conference recommend adjuvant chemotherapy for TNBC [26], although a specific regimen for such adjuvant treatment has yet to be presented. In the 190 TNBC cases of the present study, patients undergoing surgery plus adjuvant therapy had a more favorable prognosis than those receiving surgery alone, only among those with Stage II disease, suggesting that adjuvant therapy is indeed useful for TNBC patients as the NCCN recommends, and is most relevant at Stage II. In the adjuvant therapy group, both univariate and multivariate analysis showed no significant difference in prognosis between the anthracyclin-based regimen and 5FU-based regimen, although patients with the former regimen showed a trend-level improvement in prognosis over those with the latter. Larger studies might be necessary to clarify the prognoses of anthracyclin-based regimen and 5FU-based regimen.

Since reliable parameters to predict the chemosensitivity of TNBC have not been clinically available, the prognostic value of E-cadherin, Ki67 and p53 expression for the outcome of adjuvant chemotherapy was evaluated. In the adjuvant chemotherapy group, the prognosis of E-cadherin-negative and Ki67-positive patients was significantly worse than that of either E-cadherin-positive or Ki67-negative patients at all stages. Taking these results together, a multivariate logistic regression analysis showed that the E-cadherin-negative and Ki67-positive expression was significantly correlated with overall survival of patients receiving adjuvant chemotherapy. Ki67 is a candidate predictive marker for chemosensitivity in all types of breast cancer [16,26]; however, Ki67 alone is not an independent prognostic factor for TNBC with adjuvant chemotherapy. These findings suggested that the combination of E-cadherin and Ki67 expression could be of predictive value for TNBC patients treated by the adjuvant chemotherapeutic regimen, only at Stage II. On the other hand, in the group undergoing surgery alone, no significant difference of prognosis was found between E-cadherin-negative and Ki67-positive patients (*n *= 17) and either E-cadherin-positive or Ki67-negative patients (*n *= 35). Since the combination of E-cadherin and Ki67 status might be a useful prognostic marker for adjuvant chemotherapy in TNBC patients, the adjuvant chemotherapy might have led to a better prognosis for the 35 cases with both E-cadherin-positive and Ki67-negative expression. In contrast, no significant difference in prognosis was found between the E-cadherin-negative and Ki67-positive cases undergoing surgery plus adjuvant chemotherapy and surgery alone cases, which might suggest that the development of new adjuvant treatment approaches is necessary for the E-cadherin-negative and Ki67-positive TNBC patients.

The Ki67 expression level was significantly high in Stages II and III TNBC tumors (63%, *P *= 0.013). These findings suggested that tumor cells at an advanced stage might have higher proliferative activity than those at an early stage.

The mechanisms responsible for the chemosensitivity of TNBC with E-cadherin-negative and Ki67-positive expression remain to be determined. Loss of E-cadherin induces epithelial-to-mesenchymal transition (EMT). EMT is a key step toward cancer metastasis. Ahmed *et al. *reported the close relationship between EMT and the cancer stem cell-like phenotype in response to chemoresistance [39]. Also, other studies have shown that Snail, Slug and Notch signaling, as EMT markers, were correlated with chemoresistance. These findings suggested that one of the possible mechanisms by which chemosensitivity is reduced in patients with TNBC with loss of E-cadherin expression may involve EMT signaling [22,37]. In contrast, several studies have reported that E-cadherin-dependent intercellular adhesion enhances chemoresistance [40-42]. The function of E-cadherin in the efficacy of chemotherapy is controversial. Further studies of the correlation between E-cadherin and chemosensitivity in TNBC might be necessary. Previous studies have demonstrated correlations between Ki67 expression and malignancy as well as patient outcomes [43,44]. Ki67 is one of the markers for chemosensitivity in breast carcinomas, but little correlation has been revealed between Ki67 expression and chemosensitivity in the triple-negative phenotype. In this study, we found that Ki67 expression had a prognostic value for the outcome of adjuvant chemotherapy in TNBC when it was combined with E-cadherin expression, but that Ki67 alone was not an independent prognostic factor.

CR Kennedy *et al. *reported that patients with TNBC who underwent adjuvant chemotherapy were less likely to die compared with those who received neoadjuvant chemotherapy or no chemotherapy. In contrast, our study reported that the adjuvant therapy is beneficial for Stage II TNBC patients. Moreover, we suggested that the combination of E-cadherin and Ki67 status might be a useful prognostic marker indicating the need for adjuvant chemotherapy in Stage II TNBC patients. These findings are novel in our study regarding the advantages of adjuvant chemotherapy. TNBC patients have a greater risk of distant metastasis [45,46]. The presence of micrometastasis in the bone marrow at the time of diagnosis of breast cancer is associated with a high risk of relapse and a poor prognosis [47,48]. Patients with bone marrow micrometastasis had tumors with a higher stage as defined by tumor size and lymph-node status, and hormone receptor-negative tumors [48-50]. These findings suggested that the observed survival benefit of adjuvant chemotherapy at Stage II may be a result of the decreased opportunity for systemic tumor shedding and growth of systemic micrometastases. Therefore, the benefit of primary tumor removal followed by adjuvant therapy may be clinically relevant for the TNBC at Stage II.

No significant difference of overall survival was found between the surgery alone group and the surgery plus adjuvant chemotherapy group at Stages I and III, while the surgery plus adjuvant therapy showed a better prognosis than the surgery alone (Additional file 1). The recurrence rate of patients at Stage I was low and the sample size of patients at Stage III was small (*n *= 24) in this study, which might suggest one of the reasons why findings may be limited to Stage II. A larger number of TNBC patients with Stages I and III might be necessary to clarify whether patients with Stages I and III also have these advantages by adjuvant chemotherapy.

The p53 expression was positive in 118 (62%) of 190 cases of TNBC in the present study, which was similar to previous reports of the p53 expression rate (42% to 56%) in TNBC [8,32,51]. p53 status was not a specific prognostic factor in TNBC patients treated by adjuvant chemotherapy. There have been many studies on the predictive role of p53 for patients treated with anthracyclines, but most of these studies were performed in a neoadjuvant setting, and thus the value of p53 for predicting the efficacy of chemotherapy remains a matter of controversy [27,28,30]. Chae *et al. *reported that p53 status was a specific prognostic factor in 135 TNBC patients treated with an adjuvant anthracycline-based regimen, although, as the authors pointed out, the sample size was small [32]. Two of the main explanations for the discrepancies among these studies are that different methods were used to assess the p53 status, and the groups were different in terms of the patient characteristics and drug regimens. Because our study was also heterogeneous in terms of the chemotherapy regimen, a larger sample size with a single regimen might be necessary to evaluate the clinical significance of p53 for TNBC patients with adjuvant chemotherapy.

## Conclusions

In conclusion, adjuvant therapy is beneficial for TNBC patients, only at Stage II. The combination of E-cadherin and Ki67 status might be a useful prognostic marker indicating the need for adjuvant chemotherapy in Stage II TNBC patients.

## Abbreviations

AC: adriamycin+cyclophosphamide; CAF: cyclophosphamide+adriamycin+fluorouracil; CEP 17: centromere 17; CMF: cyclophosphamide+methotorexate+fluorouracil; EC: epirubicin+cyclophosphamide; EMT: epithelial-to-mesenchymal transition; ER: estrogen receptor; FEC: fluorouracil+epirubicin+cyclophosphamide; FISH: fluorescent *in situ *hybridization; HER2: human epidermal growth factor receptor 2; IHC: Immunohistochemistry; NCCN: National Comprehensive Cancer Network; PR: progesterone receptor; TNBC: Triple-negative breast cancer; UFT: tegafur-uracil; 5'DFUR: doxifluridine; 5FU: 5-fluorouracil.

## Competing interests

The authors declare that they have no competing interests.

## Authors' contributions

SK participated in study concepts, study design, data analysis and interpretation, and prepared and edited the manuscript. MY participated in study concepts, study design, quality control of data and algorithms, and in the preparation, editing and review of the manuscript. TT took part in data acquisition and statistical analysis. NA participated in data analysis and interpretation, and statistical analysis. KI and YO took part in data acquisition. TI and KH reviewed the manuscript. All authors read and approved the final manuscript

## Supplementary Material

Additional file 1 **File showing overall survival of patients at Stages I and III according to receipt of adjuvant therapy**. When restricting the analysis to patients with Stages I **(A) **and III **(B) **cancers, the overall survival of the surgery plus adjuvant chemotherapy group was not significantly better than that of the surgery alone group. Click here for file

Additional file 2 **File showing survival of patients at Stages I and III based on E-cadherin expression and Ki67 expression**. **(A) **The prognosis of the combination of E-cadherin-negative and Ki67-positive expression cancer patients was significantly poorer than that of the combination of E-cadherin-positive and Ki67-negative expression cancer patients at Stage I (*P *= 0.0437). **(B) **In contrast, no significant difference was found at Stage III. Click here for file

Additional file 3 **File showing significance of E-cadherin and Ki67 expression in patients with or without adjuvant therapy**. In surgery plus adjuvant chemotherapy group, the overall survival of TNBC patients having both E-cadherin-negative and Ki67-positive expression was significantly worse than that of patients with E-cadherin-positive and Ki67-negative at Stages I and III. In contrast, no significant difference was found in surgery alone group. Click here for file
