# Supplementary material for: Advantages of adjuvant chemotherapy for patients with triple-negative breast cancer at Stage II: usefulness of prognostic markers E-cadherin and Ki67
Source: Breast Cancer Res. 2011 Nov 30;13(6):R122. doi: 10.1186/bcr3068 (PMC3326564; doi:10.1186/bcr3068)
Supplement: Additional file 1 — File showing overall survival of patients at Stages I and III according to receipt of adjuvant therapy. When restricting the analysis to patients with Stages I (A) and III (B) cancers, the overall survival of the surgery plus adjuvant chemotherapy group was not significantly better than that of the surgery alone group. [file bcr3068-S1.PPT]

## Slide 1
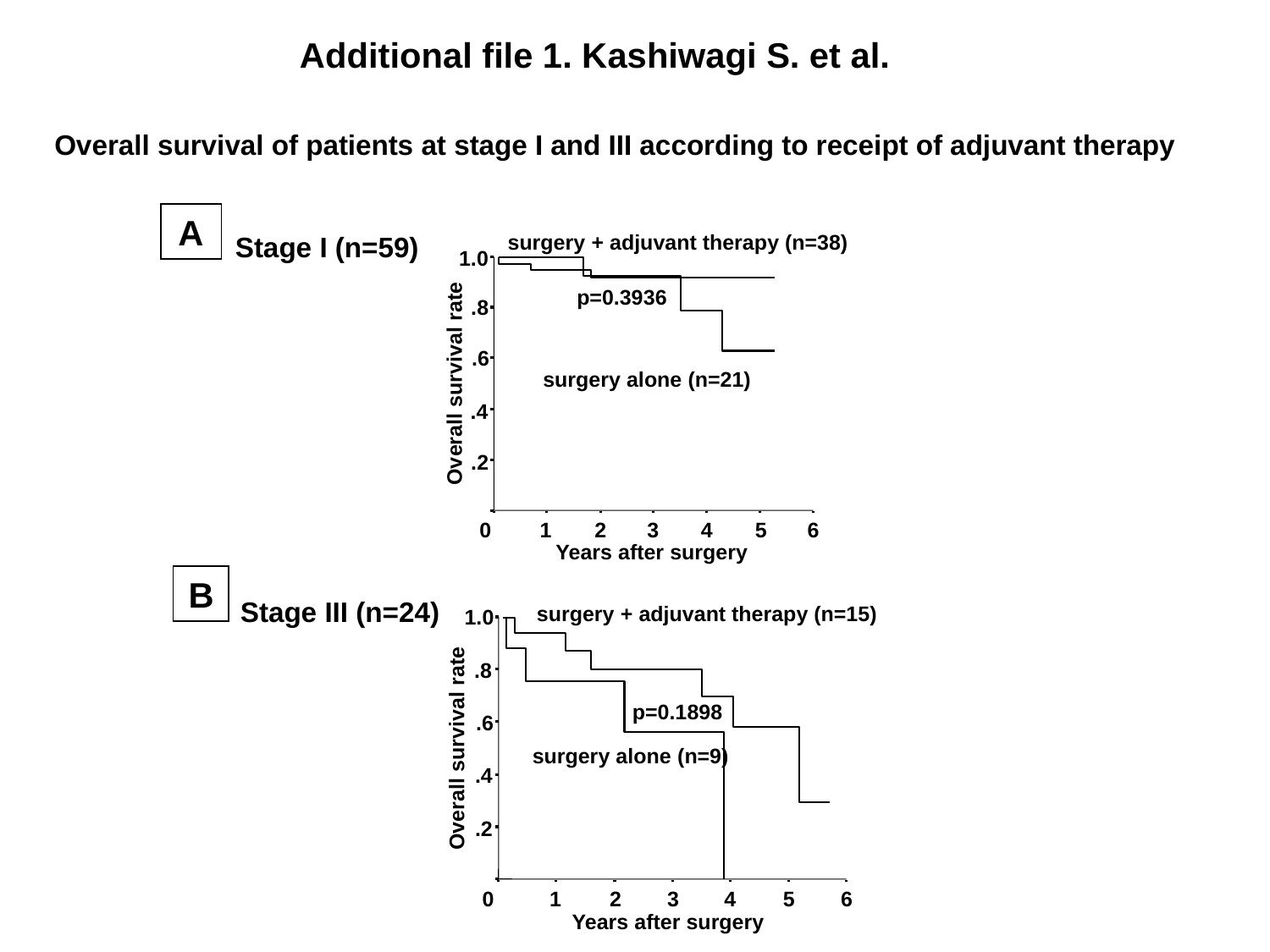

Additional file 1. Kashiwagi S. et al.
Overall survival of patients at stage I and III according to receipt of adjuvant therapy
A
surgery + adjuvant therapy (n=38)
Stage I (n=59)
1.0
p=0.3936
.8
.6
surgery alone (n=21)
Overall survival rate
.4
.2
0
1
2
3
4
5
6
Years after surgery
B
Stage III (n=24)
surgery + adjuvant therapy (n=15)
1.0
.8
p=0.1898
.6
Overall survival rate
surgery alone (n=9)
.4
.2
0
1
2
3
4
5
6
Years after surgery
