# Supplementary material for: Advantages of adjuvant chemotherapy for patients with triple-negative breast cancer at Stage II: usefulness of prognostic markers E-cadherin and Ki67
Source: Breast Cancer Res. 2011 Nov 30;13(6):R122. doi: 10.1186/bcr3068 (PMC3326564; doi:10.1186/bcr3068)
Supplement: Additional file 2 — File showing survival of patients at Stages I and III based on E-cadherin expression and Ki67 expression. (A) The prognosis of the combination of E-cadherin-negative and Ki67-positive expression cancer patients was significantly poorer than that of the combination of E-cadherin-positive and Ki67-negative expression cancer patients at Stage I (P = 0.0437). (B) In contrast, no significant difference was found at Stage III. [file bcr3068-S2.PPT]

## Slide 1
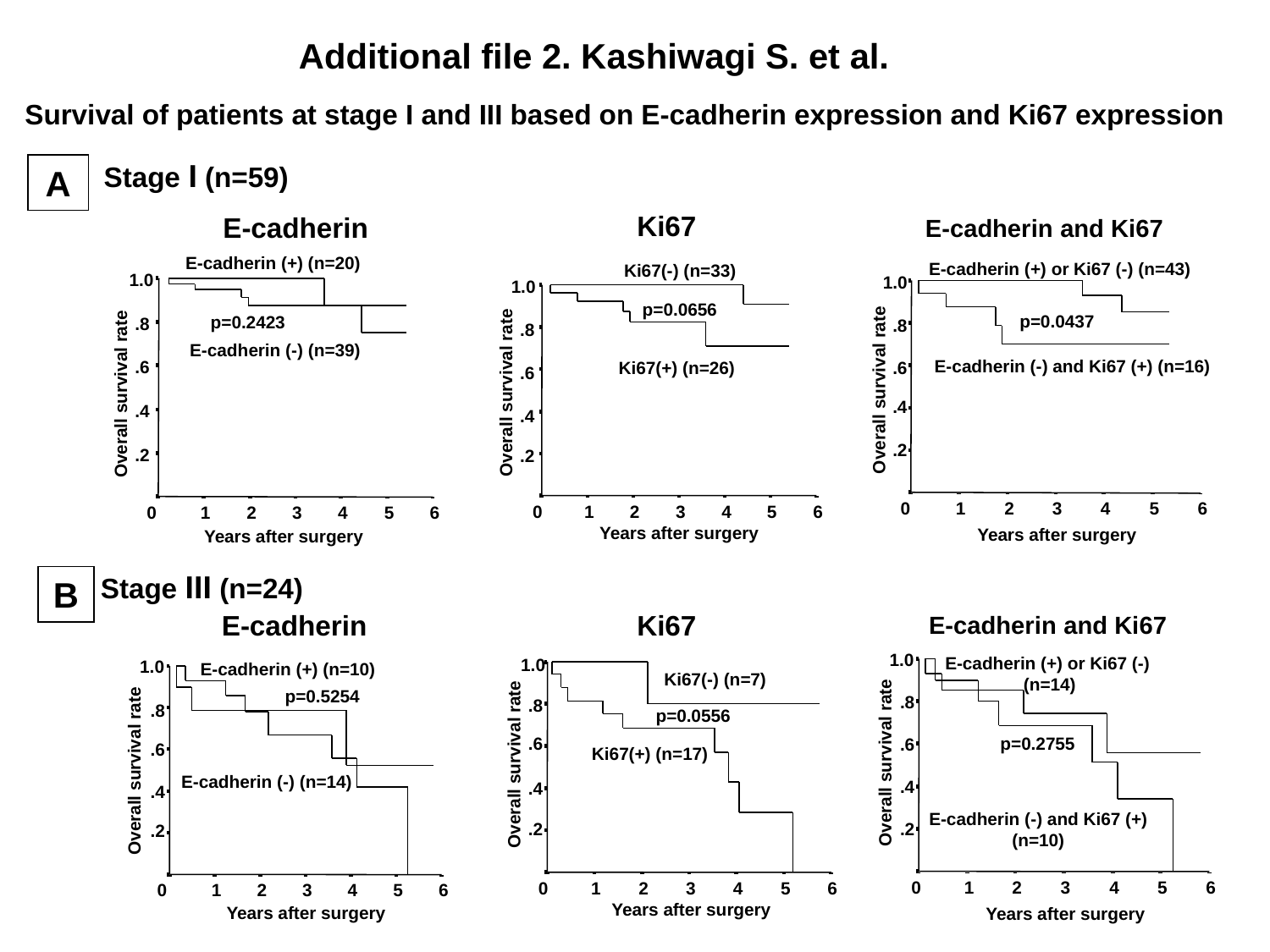

Additional file 2. Kashiwagi S. et al.
Survival of patients at stage I and III based on E-cadherin expression and Ki67 expression
Stage I (n=59)
A
Ki67
E-cadherin
E-cadherin and Ki67
E-cadherin (+) (n=20)
E-cadherin (+) or Ki67 (-) (n=43)
Ki67(-) (n=33)
1.0
1.0
1.0
p=0.0656
p=0.0437
p=0.2423
.8
.8
.8
E-cadherin (-) (n=39)
E-cadherin (-) and Ki67 (+) (n=16)
Ki67(+) (n=26)
.6
.6
.6
Overall survival rate
Overall survival rate
Overall survival rate
.4
.4
.4
.2
.2
.2
0
1
2
3
4
5
6
0
1
2
3
4
5
6
0
1
2
3
4
5
6
Years after surgery
Years after surgery
Years after surgery
Stage III (n=24)
B
E-cadherin
Ki67
E-cadherin and Ki67
E-cadherin (+) or Ki67 (-)
(n=14)
1.0
E-cadherin (+) (n=10)
1.0
1.0
Ki67(-) (n=7)
p=0.5254
.8
.8
p=0.0556
.8
p=0.2755
.6
.6
Ki67(+) (n=17)
.6
Overall survival rate
Overall survival rate
Overall survival rate
E-cadherin (-) (n=14)
.4
.4
.4
E-cadherin (-) and Ki67 (+) (n=10)
.2
.2
.2
0
1
2
3
4
5
6
0
1
2
3
4
5
6
0
1
2
3
4
5
6
Years after surgery
Years after surgery
Years after surgery
