# Supplementary material for: Advantages of adjuvant chemotherapy for patients with triple-negative breast cancer at Stage II: usefulness of prognostic markers E-cadherin and Ki67
Source: Breast Cancer Res. 2011 Nov 30;13(6):R122. doi: 10.1186/bcr3068 (PMC3326564; doi:10.1186/bcr3068)
Supplement: Additional file 3 — File showing significance of E-cadherin and Ki67 expression in patients with or without adjuvant therapy. In surgery plus adjuvant chemotherapy group, the overall survival of TNBC patients having both E-cadherin-negative and Ki67-positive expression was significantly worse than that of patients with E-cadherin-positive and Ki67-negative at Stages I and III. In contrast, no significant difference was found in surgery alone group. [file bcr3068-S3.PPT]

## Slide 1
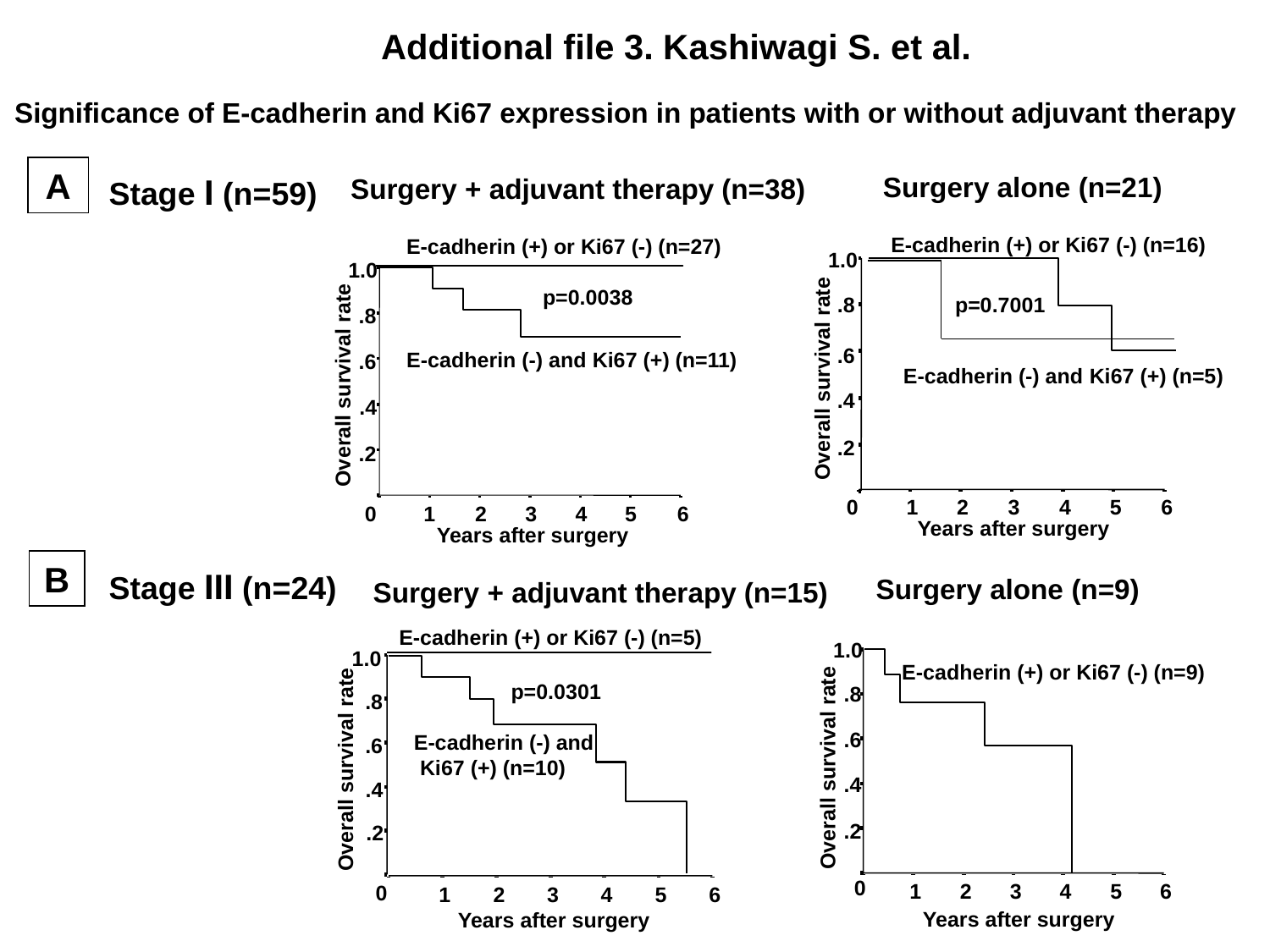

Additional file 3. Kashiwagi S. et al.
Significance of E-cadherin and Ki67 expression in patients with or without adjuvant therapy
A
Surgery alone (n=21)
Stage I (n=59)
Surgery + adjuvant therapy (n=38)
E-cadherin (+) or Ki67 (-) (n=16)
E-cadherin (+) or Ki67 (-) (n=27)
1.0
1.0
p=0.0038
p=0.7001
.8
.8
E-cadherin (-) and Ki67 (+) (n=11)
.6
.6
E-cadherin (-) and Ki67 (+) (n=5)
Overall survival rate
Overall survival rate
.4
.4
.2
.2
0
1
2
3
4
5
6
0
1
2
3
4
5
6
Years after surgery
Years after surgery
B
Stage III (n=24)
Surgery alone (n=9)
Surgery + adjuvant therapy (n=15)
E-cadherin (+) or Ki67 (-) (n=5)
1.0
1.0
E-cadherin (+) or Ki67 (-) (n=9)
p=0.0301
.8
.8
E-cadherin (-) and
 Ki67 (+) (n=10)
.6
.6
Overall survival rate
Overall survival rate
.4
.4
.2
.2
0
1
2
3
4
5
6
0
1
2
3
4
5
6
Years after surgery
Years after surgery
